# Evaluating The Impact of Ring Augmentation In Sleeve Gastrectomy: A Retrospective Propensity-weighted Cohort Study

**DOI:** 10.1186/s12876-025-04476-9

**Published:** 2025-12-02

**Authors:** Mohamed Hany, Islam Mohamed Awad Sedik, Medhat Anwar, Rabie Abd Elwahed, Alaa Hamza, Mohamed Samir

**Affiliations:** 1 Department of Experimental and Clinical Surgery, Medical Research Institute, Alexandria University, Alexandria , Egypt; 2 Bariatric Surgery Unit, Madina Women’s Hospital, Alexandria, Egypt; 3 Department of Surgery, Medical Research Institute, Alexandria University, 165 Horreya Avenue, Hadara, 21561 Alexandria Egypt

**Keywords:** Laparoscopic sleeve gastrectomy, Ring-Augmented sleeve gastrectomy, Non-Ring-Augmented laparoscopic sleeve gastrectomy, Augmented ring, Obesity

## Abstract

**Background:**

Sleeve gastrectomy (SG) is the most commonly performed metabolic surgery for obesity but is associated with long-term recurrent weight gain (RWG) and recurrence of comorbidities. Ring-augmented sleeve gastrectomy (RASG) has been proposed to improve weight durability by preventing gastric dilation and loss of restriction.

**Methods:**

This retrospective propensity-weighted cohort study compared outcomes of RASG and non-ring-augmented SG (NRASG) over two years in patients with morbid obesity. A total of 1,392 SG cases performed between 2021 and 2022 were reviewed, and after propensity score weighting, 132 RASG and 125 NRASG patients were analyzed.

**Results:**

RASG was associated with significantly higher total and excess weight loss at two years (TWL: 47.4% vs. 38.5%; EWL: 102.8% vs. 82.0%; *p* < 0.001) and a complete absence of RWG, compared to a 5.6% recurrence rate in the NRASG group. Comorbidity resolution rates and nutritional deficiencies were similar across groups, while ring-related complications were rare and manageable. Endoscopic evaluation at one year showed no significant difference in GERD incidence.

**Conclusion:**

These findings suggest that RASG provides more durable weight loss without added morbidity compared to NRASG. Further prospective studies incorporating patient-reported outcomes and physiologic assessments are warranted to validate long-term benefits and safety.

**Supplementary Information:**

The online version contains supplementary material available at 10.1186/s12876-025-04476-9.

## Introduction

 The global escalation of obesity has reached epidemic proportions, imposing significant health burdens and contributing to a spectrum of associated diseases that reduce both life expectancy and quality of life [1]. In response, Metabolic and Bariatric Surgery (MBS) has emerged as the most effective long-term intervention for achieving substantial and sustained weight loss, particularly among patients with severe obesity. Among the various surgical options, sleeve gastrectomy (SG) has become the most frequently performed MBS procedure worldwide, accounting for over half of all primary operations [2, 3]. Its growing popularity is attributed to its relative technical simplicity, favorable safety profile, and effectiveness in promoting weight loss and improving obesity-associated comorbidities [3–7].

SG effectively curtails food intake and diminishes ghrelin secretion, facilitating weight loss [8, 9]. However, SG is not devoid of challenges, with one of the most pressing issues being the propensity for long-term recurrent weight gain (RWG). This is particularly pronounced in patients with a baseline body mass index (BMI) greater than 40 kg/m². Literature indicates that RWG rates can vary significantly, ranging from 14% to 37% five years post-operatively. This phenomenon is often linked to factors such as gastric pouch dilation, loss of restrictive capacity, and insufficient adherence to prescribed dietary and lifestyle modifications [10–13].

To mitigate the risk of RWG and reinforce the restrictive component of SG, Ring-Augmented Sleeve gastrectomy (RASG) was introduced, which involves the placement of a non-adjustable gastric ring around the gastric sleeve to prevent dilation and endorse satiety over time [14, 15]. Early studies suggest that RASG may enhance long-term excess weight loss (EWL), reduce RWG, and potentially improve metabolic outcomes, including glycemic control and hypertension resolution, beyond what is typically achieved with conventional SG [7, 16]. Moreover, Recent mid-term outcomes support this notion [17].

Additionally, emerging evidence highlights the potential of RASG in attenuating the incidence and severity of gastroesophageal reflux disease (GERD), a notable postoperative complication of standard SG. By maintaining the integrity of the gastric sleeve and preventing pouch dilation, the ring may reduce intra-gastric pressure and reflux symptoms, thereby improving both patient outcomes and quality of life [17–19].

However, despite its theoretical and early clinical advantages, RASG is not without potential drawbacks. Ring-related complications, including slippage, erosion, and obstruction, remain a concern, necessitating close postoperative monitoring and, in some cases, surgical intervention [20], necessitating high technical demands [21]. Moreover, questions remain regarding the long-term nutritional impact, adherence to postoperative care, and psychological readiness of patients undergoing this more restrictive modification of SG [14, 18, 22].

In this context, the evaluation of RASG compared to traditional non-ring-augmented sleeve gastrectomy (NRASG) warrants further clinical investigation, particularly regarding durability of weight loss, prevention of RWG, and resolution of associated diseases. A comprehensive, evidence-based comparison may inform surgical decision-making and optimize long-term outcomes for patients with morbid obesity.

This retrospective cohort study evaluates the long-term outcomes of RASG using the MiniMizer Gastric Ring^®^ compared to conventional NRASG in patients with morbid obesity. Primary endpoints include excess and total weight loss, recurrent weight gain, and resolution of obesity-related comorbidities over a two-year follow-up. Secondary outcomes focus on procedural safety, including nutritional deficiencies and ring-related complications. The study aims to determine whether ring augmentation enhances weight loss durability without compromising safety.

## Methods

### Study design and setting

This retrospective cohort study was conducted at the Medical Research Institute, Alexandria University, Egypt, and Madina Women’s Hospital. Data were obtained from a prospectively maintained institutional electronic database of MBS procedures, including standardized preoperative assessment records, intraoperative details, and postoperative follow-up.

Although the surgical team and operative protocols are consistent with those described in our previous publication evaluating mid-term outcomes of Ring Augmented and non-Ring Augmented SG [17], the present cohort is entirely distinct. It includes a new patient population operated during a later time frame, using updated data collection tools and follow-up protocols.

Ethical approval for this study was granted by the Institutional Review Board of the Medical Research Institute, Alexandria University (IRB00010526; Approval No. E/C. S/N. R22/2024). All participants provided written informed consent for the use of their anonymized clinical data in research.

### Patient selection

The study included adult patients who underwent sleeve gastrectomy (SG) between January 2021 and December 2022. Patients were grouped based on whether they received a Ring-Augmented Sleeve Gastrectomy (RASG) using a MiniMizer Gastric Ring^®^ or a conventional Non-Ring-Augmented Sleeve Gastrectomy (NRASG). All procedures were performed by the same experienced bariatric surgical team following uniform operative protocols. Ethical approval was obtained from the institutional review board, and written informed consent was collected for data use in research.

Prior to surgery, patients received standardized counseling on both conventional and ring-augmented techniques. This session detailed the benefits of ring augmentation, including enhanced durability of weight loss and decreased risk of RWG, as well as potential risks like intolerance, dysphagia, and ring-related complications. Patients then participated in a shared decision-making process to select their preferred procedure.

Following NIH guidelines [23], the inclusion criteria were adults aged 18–60 with a BMI ≥ 40 kg/m² or ≥ 35 kg/m² with obesity-related diseases. Exclusion criteria included previous MBS or upper gastrointestinal surgery, pregnancy or lactation during follow-up, active malignancy, severe psychiatric illness, or systemic conditions compromising follow-up compliance. Only patients with complete follow-up data throughout the 24 months were included in the final analyses.

### Preoperative evaluation

Patients underwent a comprehensive preoperative workup including history, physical exam, anthropometric measurements, and laboratory tests. These assessed glucose homeostasis, lipid profile, renal and liver function, thyroid hormones, and nutritional markers (vitamin B12, vitamin D, calcium, ferritin, albumin). Esophagogastroduodenoscopy (EGD) and abdominal ultrasound were routinely performed to assess for structural abnormalities or GERD.

### Surgical technique

All patients underwent laparoscopic SG using a standardized technique under general anesthesia. For patients in the NRASG group, a five-port laparoscopic approach was used: three 12-mm ports (camera, right-hand, and left-hand working ports) and two 5-mm ports (liver retractor and assistant). Pneumoperitoneum was established using optical trocar entry. The greater omentum was dissected off the greater curvature of the stomach, starting 4–5 cm proximal to the pylorus and proceeding toward the angle of His, using the EnSeal device (Ethicon Endo-Surgery, Cincinnati, OH, USA). Posterior gastric adhesions were divided, and Belsey’s pad of fat was routinely excised for optimal fundus exposure.

Gastric division was performed over a 40-Fr calibration bougie using an Echelon Flex Endopath 60-mm linear stapler (Ethicon Endo-Surgery), with green, gold, and blue reloads selected according to tissue thickness. The resection extended from 3 to 5 cm proximal to the pylorus up to the angle of His. To reinforce the staple line, running seromuscular sutures were applied using absorbable 3–0 V-Loc™ 180 barbed sutures (Covidien, Mansfield, MA, USA). In patients with an incidental intraoperative hiatal hernia, posterior cruroplasty was performed before gastric resection using non-absorbable barbed sutures.

The RASG procedure followed the same steps as NRASG. After sleeve creation, a non-adjustable MiniMizer Gastric Ring^®^ (7.5 cm circumference; 1.75 cm internal diameter, Bariatric Solutions, Switzerland) was introduced and placed 4–5 cm below the gastroesophageal junction around the proximal gastric sleeve. The ring was loosely positioned to avoid excessive tension and secured with non-absorbable sutures passed through the ring’s eyelets to prevent migration or slippage [18, 21]. Proper ring placement aimed to preserve sleeve geometry, reinforce restriction, and potentially reduce proximal dilation and gastroesophageal reflux symptoms [18, 21]. Care was taken to avoid overtightening, which may lead to dysphagia, regurgitation, or ischemic complications [15, 20].

### Postoperative follow-up and assessments

Follow-up visits were scheduled at 6 months, 1 year, and 2 years postoperatively. At each visit, anthropometric data (weight, BMI, %EWL, %TWL), laboratory assessments for nutritional status, and resolution of comorbidities (diabetes, hypertension, dyslipidemia) were documented. Recurrent Weight Gain (RWG) was defined as an increase in BMI ≥ 35 kg/m² after initial reduction; Suboptimal weight loss (SWL) was defined as %EWL < 50% at 1 year.

Laboratory tests were repeated at each follow-up to monitor nutritional status, including indicators for anemia, hypoalbuminemia, hypocalcemia, and deficiencies in vitamin D, vitamin B12, and ferritin. Comorbidity resolution or improvement, specifically for hypertension, type 2 diabetes mellitus, and dyslipidemia, was assessed at each time point. Moreover, all patients underwent a standardized postoperative esophagogastroduodenoscopy (EGD) at 1 year to assess for GERD, hiatal hernia, sleeve stricture, or ring-related complications.

### Nutritional supplementation protocol

Postoperative nutritional supplementation was standardized across both groups. All patients received oral multivitamins, calcium with vitamin D, vitamin B12, and iron as per institutional guidelines, with adherence monitored during follow-up.

### Sample size and outcome measures

The sample size calculation was done using R software version 4.2.2 and its “WebPower” package. We used a medium effect size of 0.25 for two-way repeated measures ANOVA, a power of 80% with a significance level of 0.05; this resulted in a minimum total sample size of 155 patients.

### Statistical analysis

All statistical analyses were conducted using R (The R Project for Statistical Computing, Vienna, Austria). Continuous variables were summarized as means ± standard errors (SE) and compared between groups using independent-samples t-tests. Categorical variables were summarized as counts and percentages and compared using Pearson’s chi-square test or Fisher’s exact test, as appropriate. Test statistics are reported with corresponding p-values. A two-sided *p* < 0.05 was considered statistically significant.

To address baseline differences between the RASG and NRASG groups, inverse probability score weighting (IPSW) was applied using the Toolkit for Weighting and Analysis of Nonequivalent Groups (Twang) package. Propensity scores were estimated via a generalized boosted model (GBM) using 5000 iterations and included a comprehensive set of preoperative covariates: age, sex, BMI, waist circumference, height, weight, and the presence of comorbidities (hypertension, diabetes, dyslipidemia, obstructive sleep apnea, osteoarthritis, gastroesophageal reflux, hiatal hernia, gallstones, menstrual irregularities, cardiac disease, psychiatric disorders, vascular complications, neoplasms, alcohol use, and smoking status). Although baseline covariates were not statistically different between groups (*p* > 0.05), the large sample size imbalance (RASG *n* = 132 vs. NRASG *n* = 1,260) and the nonrandom allocation necessitated adjustment to reduce residual confounding and ensure exchangeability. The average treatment effect on the treated (ATT) framework was used to compare the outcomes of patients who underwent RASG with a weighted sample of patients who underwent NRASG.

Covariate balance before and after weighting was assessed using absolute standardized mean differences (SMDs) and p-values from t-tests. Covariates with SMDs < 0.10 after weighting were considered adequately balanced. These results are summarized in Supplementary Figures S1 and S2. The weighted sample characteristics are shown in Table 2.

Weight loss outcomes, including weight, BMI, percent excess weight loss (%EWL), and percent total weight loss (%TWL), were analyzed over time using generalized estimating equations (GEE) with an exchangeable correlation structure. GEE models provided estimated means and 95% confidence intervals (CI) at each time point, as well as between-group differences. Binary outcomes such as Suboptimal weight Loss (SWL) and Recurrent weight Gain (RWG) were compared using Fisher’s exact test. All models accounted for the weighting structure to maintain valid inference.

## Results

### Study population and baseline characteristics

This study included a retrospective evaluation of 1,392 patients who underwent SG between January 2021 and December 2022. Among them, 132 patients underwent Ring-Augmented Sleeve Gastrectomy (RASG) and 1,260 patients received Non-Ring-Augmented Sleeve Gastrectomy (NRASG) (Table 1). No significant differences were observed in demographics (age, sex), anthropometrics (waist circumference, weight, BMI), preoperative labs, or comorbidities (hypertension, diabetes, dyslipidemia, OSA, psychiatric or cardiac conditions, etc.), indicating well-matched cohorts before adjustment.

**Table 1:** Baseline characteristics of patients undergoing Ring-Augmented (RASG) and Non-Ring augmented sleeve gastrectomy (NRASG) before propensity score Weighting. Data are presented as mean ± SE or n (%). Although no statistically significant differences were observed between groups (all *p* > 0.05), inverse probability score weighting (IPSW) was subsequently applied to adjust for the large sample size imbalance and potential residual confounding

Variable	RASG (*n* = 132)	NRASG (*n* = 1260)	Test statistic	*p*
**Demographics**				
Sex(female)	104 (78.8)	939 (74.5)	χ² = 1.16	0.332
Age	34.4 ± 0.8	35.5 ± 0.3	t = −1.20	0.233
**Obesity-related Diseases and Other Comorbidities**				
Hypertension	40 (30.3)	381 (30.2)	χ² = 0.00	1.000
Diabetes	24 (18.2)	218 (17.3)	χ² = 0.06	0.894
Dyslipidemia	40 (30.3)	379 (30.1)	χ² = 0.00	1.000
Sleep apnea	19 (14.4)	160 (12.7)	χ² = 0.31	0.677
Osteoarthritis	34 (25.8)	322 (25.6)	χ² = 0.00	1.000
Gallstones	13 (9.8)	129 (10.2)	χ² = 0.02	1.000
Cardiac Disease	8 (6.1)	80 (6.3)	χ² = 0.02	1.000
Psychiatric Disorder	16 (12.1)	162 (12.9)	χ² = 0.06	0.917
Vascular Disorder	20 (15.2)	191 (15.2)	χ² = 0.00	1.000
Neoplasm	2 (1.5)	23 (1.8)	Fisher	1.000
Smoking	47 (35.6)	445 (35.3)	χ² = 0.00	1.000
Alcohol	2 (1.5)	18 (1.4)	Fisher	1.000
Menstrual Abnormalities	11 (8.7)	75 (8.0)	χ² = 0.06	0.962
History of Venous Thromboembolism (DVT/PE)	2 (1.5)	20 (1.6)	Fisher	1.000
**Preoperative Endoscopic Assessment**				
Hiatal hernia	7 (5.3)	78 (6.2)	χ² = 0.16	0.830
Esophagitis	25 (18.9)	254 (20.2)	χ² = 0.11	0.827
**Anthropometrics**				
Waist (cm)	119.7 ± 1.4	119.5 ± 0.5	t = 0.11	0.911
Weight (kg)	133.1 ± 2.3	133.2 ± 0.7	t = −0.05	0.958
BMI (kg/m^2^)	47.7 ± 0.6	47.7 ± 0.2	t = 0.04	0.972
**Preoperative labs**				
Hb (mg/dl)	12.9 ± 0.1	12.9 ± 0.0	t = 0.17	0.864
WBC (10^9^/L)	7.4 ± 0.2	7.4 ± 0.1	t = 0.26	0.797
AST (U/L)	22.0 ± 0.7	22.1 ± 0.2	t = −0.04	0.965
ALT (U/L)	28.8 ± 1.0	28.8 ± 0.3	t = 0.03	0.976
UREA (mg/dL)	28.3 ± 0.7	28.2 ± 0.2	t = 0.02	0.984
CRT (mg/dL)	0.8 ± 0.0	0.8 ± 0.0	t = 0.00	0.997
INR	1.1 ± 0.0	1.1 ± 0.0	t = 0.13	0.899
T3 (ng/dl)	2.9 ± 0.1	2.9 ± 0.0	t = 0.11	0.913
T4 (ng/dl)	1.2 ± 0.0	1.2 ± 0.0	t = −0.24	0.810
TSH (mIU/L)	2.7 ± 0.1	2.7 ± 0.0	t = 0.07	0.946
Fasting glucose (mg/dL)	101.6 ± 2.3	101.4 ± 0.7	t = 0.11	0.911
HbA1c (%)	7.9 ± 0.1	7.9 ± 0.0	t = −0.01	0.994

Continuous variables are expressed as mean ± standard error (SE). Categorical variables are expressed as count (percentage). p-values were derived from independent t-tests for continuous variables and chi-square or Fisher’s exact tests for categorical variables. Test statistics are shown as t for t-tests and χ² for chi-square tests; “Fisher” indicates Fisher’s exact test was used. No variable showed a statistically significant difference at *p* < 0.05

*DVT* Deep Vein Thrombosis, *PE* Pulmonary Embolism, *BMI* Body Mass Index, *Hb* Hemoglobin, *WBC* White Blood Cells Count, *AST* Aspartate Aminotransferase, *ALT* Alanine Aminotransferase, *CRT* Creatinine, *TSH* Thyroid Stimulating Hormone

### Propensity score weighting

To adjust for baseline imbalances, inverse probability score weighting (IPSW) was performed using a generalized boosted model incorporating 5,000 iterations. Covariates included demographic, anthropometric, and clinical variables (age, sex, BMI, waist circumference, comorbidities, etc.). The average treatment effect on the treated (ATT) framework was applied, and covariate balance was evaluated using standardized mean differences (SMDs). After weighting, all SMDs were < 0.10, confirming adequate balance (Table 2) (Supplementary File 1, Figure S1, S2).

**Table 2:** Weighted baseline characteristics of patients after application of inverse probability score weighting (IPSW). Data are presented as mean ± SE or n (%). Post-weighting, all covariates were adequately balanced (standardized mean differences < 0.10), ensuring exchangeability between groups

Variable	RASG (*n*= 132)	NRASG (*n*= 125)	Test statistic	*P*
**Demographics**				
Sex(female)	104 (78.8)	98 (78.9)	χ² = 0.00	0.968
Age	34.4 ± 0.8	34.3 ± 0.3	t = 0.10	0.924
**Obesity-related Diseases and Other Comorbidities**				
Hypertension	40 (30.3)	38 (30.2)	χ² = 0.00	0.985
Diabetes	24 (18.2)	21 (17.1)	χ² = 0.09	0.761
Dyslipidemia	40 (30.3)	37 (30.0)	χ² = 0.00	0.947
Sleep apnea	19 (14.4)	17 (13.9)	χ² = 0.03	0.873
Osteoarthritis	34 (25.8)	31 (24.9)	χ² = 0.05	0.831
Gallstones	13 (9.8)	12 (9.7)	χ² = 0.00	0.952
Cardiac Disease	8 (6.1)	8 (6.4)	χ² = 0.02	0.887
Psychiatric Disorder	16 (12.1)	16 (12.6)	χ² = 0.02	0.881
Vascular Disorder	20 (15.2)	19 (15.2)	χ² = 0.00	0.979
Neoplasm	2 (1.5)	2 (1.3)	Fisher	0.862
Smoking	47 (35.6)	44 (35.2)	χ² = 0.01	0.919
Alcohol	2 (1.5)	2 (1.5)	Fisher	0.997
Menstrual Abnormalities	11 (8.7)	9 (7.4)	χ² = 0.20	0.654
History of Venous Thromboembolism (DVT/PE)	2 (1.5)	2 (1.7)	Fisher	0.859
**Preoperative endoscopic assessment**				
Hiatal hernia	7 (5.3)	7 (5.5)	χ² = 0.01	0.912
Esophagitis	25 (18.9)	24 (19.6)	χ² = 0.03	0.865
Anthropometrics				
Waist (cm)	119.7 ± 1.4	119.3 ± 0.6	t = 0.26	0.796
Weight (kg)	133.1 ± 2.3	133.1 ± 1.0	t = -0.02	0.981
BMI (kg/m^2^)	47.7 ± 0.6	47.7 ± 0.2	t = -0.01	0.993
**Preoperative labs**				
Hb (mg/dl)	12.9 ± 0.1	12.9 ± 0.1	t = 0.08	0.937
WBC (10^9^/L)	7.4 ± 0.2	7.3 ± 0.1	t = 0.67	0.504
AST (U/L)	22.0 ± 0.7	22.0 ± 0.2	t = 0.08	0.934
ALT (U/L)	28.8 ± 1.0	29.0 ± 0.4	t = -0.19	0.851
UREA (mg/dL)	28.3 ± 0.7	28.3 ± 0.3	t = -0.08	0.938
CRT (mg/dL)	0.8 ± 0.0	0.8 ± 0.0	t = -0.05	0.962
INR	1.1 ± 0.0	1.1 ± 0.0	t = 0.12	0.905
T3 (ng/dl)	2.9 ± 0.1	2.9 ± 0.0	t = 0.17	0.864
T4 (ng/dl)	1.2 ± 0.0	1.2 ± 0.0	t = -0.17	0.867
TSH (mIU/L)	2.7 ± 0.1	2.6 ± 0.0	t = 0.28	0.778
Fasting glucose (mg/dL)	101.6 ± 2.3	101.3 ± 0.9	t = 0.12	0.901
HbA1c (%)	7.9 ± 0.1	7.9 ± 0.0	t = 0.08	0.939

Continuous variables are presented as mean ± standard error (SE). Categorical variables are presented as number (percentage). p-values were calculated using independent samples t-tests for continuous variables and chi-square or Fisher’s exact tests for categorical variables, as appropriate. Test statistics are reported as t for t-tests and χ² for chi-square tests; "Fisher" indicates Fisher’s exact test was used. The groups were well balanced post-weighting, with all *p*-values >0.05 and minimal differences across all baseline characteristics

*BMI* Body Mass Index, *DVT* Deep Vein Thrombosis, *PE* Pulmonary Embolism, *BMI* Body Mass Index, *Hb *Hemoglobin, *WBC* White Blood Cells Count, *AST* Aspartate Aminotransferase, *ALT* Alanine Aminotransferase, *CRT* Creatinine, *TSH* Thyroid Stimulating Hormone

### Postoperative weight loss outcomes

**Fig. 1:**
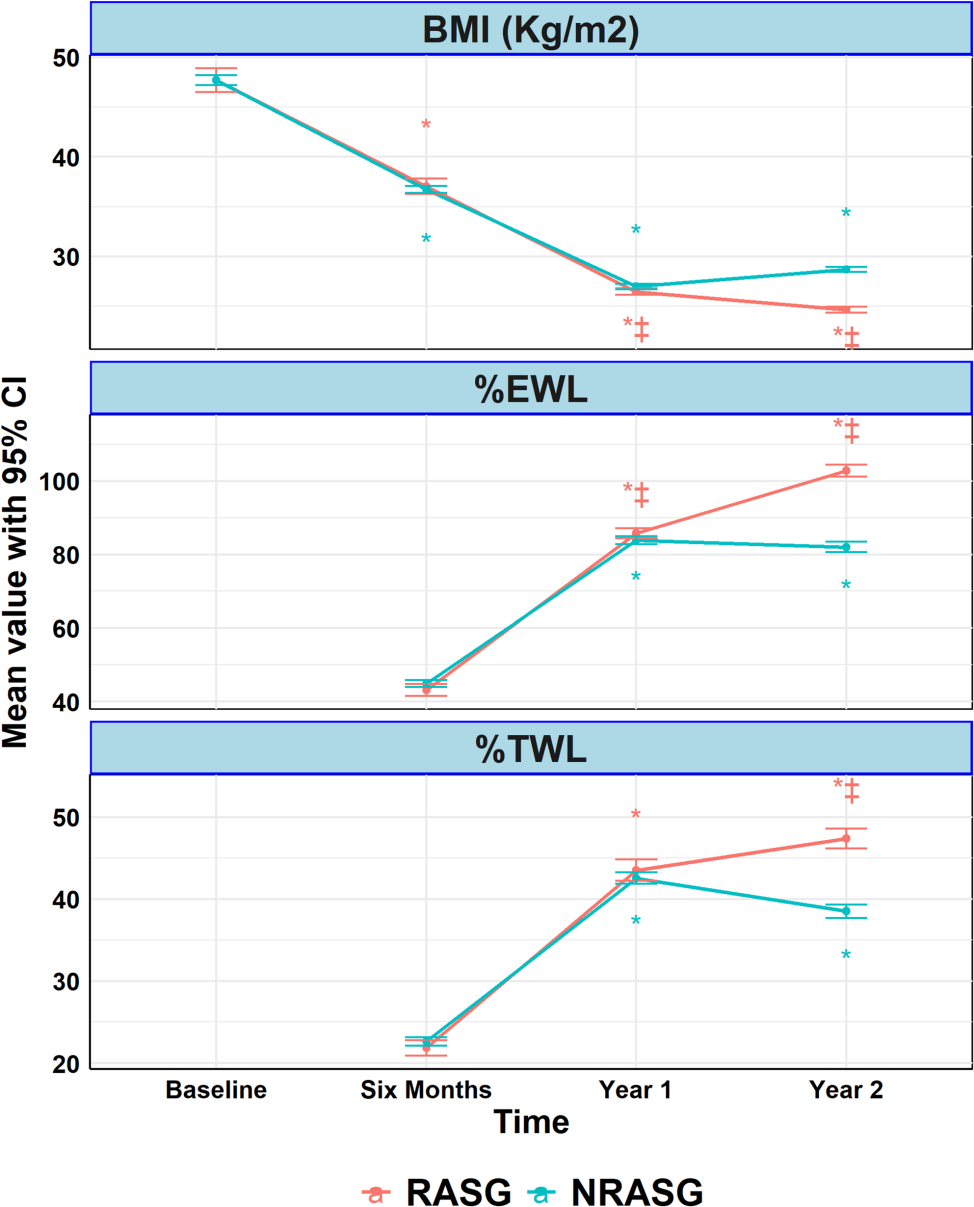
Trends in BMI, %EWL, and %TWL Over Time in RASG and NRASG Groups. Figure illustrates the changes in BMI (kg/m²), %EWL, and %TWL from baseline to two years following surgery in patients who underwent RASG or NRASG. Both groups showed marked

Using generalized estimating equation (GEE) analysis adjusted by inverse probability score weighting (IPSW) on weight loss trajectories over two years, both RASG and NRASG groups achieved substantial reductions in weight and BMI over time; however, RASG showed significantly superior long-term outcomes (Table 3) (Fig. 1).

**Table 3:** Weight loss outcomes estimated from GEE analyses within and between RASG and NRASG groups

Variable	Time	RASG			NRASG			MD between RASG and NRASG (95% CI)	*p*
		Mean ± SE	MC from baseline	*p*	Mean ± SE	MC from baseline	*p*		
Weight (kg)	Baseline	133.1 ± 2.3	Reference		133.1 ± 1.0	Reference		−0.06 (−4.91, 4.79)	0.981
	Six months	103.2 ± 1.5	−29.90 (−35.21, −24.59)	**< 0.001**	102.1 ± 0.6	−31.03 (−33.26, −28.79)	**< 0.001**	1.07 (−2.04, 4.18)	0.501
	Year 1	73.7 ± 0.8	−59.35 (−64.10, −54.60)	**< 0.001**	74.7 ± 0.4	−58.46 (−60.45, −56.46)	**< 0.001**	−0.95 (−2.69, 0.79)	0.284
	Year 2	68.7 ± 0.8	−64.34 (−69.07, −59.61)	**< 0.001**	79.2 ± 0.3	−53.92 (−55.87, −51.97)	**< 0.001**	−10.48 (−12.10, −8.86)	**< 0.001**
	SWL at year 1, n (%)	0 (0)			4 (3.3)			Fisher *p* < 0.001	
	RWG at year 2, n (%)	0 (0)			7 (5.6)			Fisher *p* < 0.001	
BMI (kg/m ^2^)	Baseline	47.7 ± 0.6	Reference		47.7 ± 0.2	Reference		−0.01 (−1.31, 1.30)	0.993
	Six months	37.1 ± 0.4	−10.64 (−12.08, −9.19)	**< 0.001**	36.7 ± 0.2	−10.97 (−11.58, −10.37)	**< 0.001**	0.33 (−0.53, 1.19)	0.452
	Year 1	26.5 ± 0.2	−21.21 (−22.47, −19.95)	**< 0.001**	27.0 ± 0.1	−20.71 (−21.25, −20.16)	**< 0.001**	−0.51 (−0.94, −0.07)	**0.022**
	Year 2	24.7 ± 0.2	−23.01 (−24.26, −21.76)	**< 0.001**	28.7 ± 0.1	−18.98 (−19.52, −18.43)	**< 0.001**	−4.04 (−4.45, −3.64)	**< 0.001**
EWL(%)	Six months	43.1 ± 0.9	Reference		44.9 ± 0.5	Reference		−1.78 (−3.71, 0.15)	0.07
	Year 1	85.7 ± 0.7	42.59 (40.45, 44.73)	**< 0.001**	83.9 ± 0.5	38.98 (37.54, 40.41)	**< 0.001**	1.83 (0.11, 3.54)	**0.037**
	Year 2	102.8 ± 0.8	59.68 (57.33, 62.03)	**< 0.001**	82.0 ± 0.7	37.09 (35.39, 38.79)	**< 0.001**	20.81 (18.64, 22.98)	**< 0.001**
TWL(%)	Six months	21.9 ± 0.5	Reference		22.6 ± 0.3	Reference		−0.79 (−1.88, 0.30)	0.154
	Year 1	43.5 ± 0.7	21.66 (20.04, 23.27)	**< 0.001**	42.6 ± 0.3	19.92 (19.07, 20.76)	**< 0.001**	0.95 (−0.51, 2.41)	0.202
	Year 2	47.4 ± 0.6	25.50 (23.95, 27.05)	**< 0.001**	38.5 ± 0.4	15.87 (14.89, 16.85)	**< 0.001**	8.84 (7.37, 10.32)	**< 0.001**

Continuous outcomes are presented as Mean ± Standard Error (SE), within-group mean change (MC) from baseline with 95% Confidence Interval (CI), and between-group mean difference (MD, RASG − NRASG). Categorical comparisons (SWL, RWG) are shown as number (%). P-values reflect significance from GEE models for continuous outcomes (with exchangeable correlation structure) and Fisher’s exact test for binary comparisons. Statistical significance was set at *p* < 0.05

*Abbreviations*: *RASG *Ring augmented Laparoscopic Sleeve Gastrectomy, *NRASG *Laparoscopic Sleeve Gastrectomy, *BMI *Body Mass Index, *EWL *Excess Weight Loss, *TWL * Total Weight Loss, *SWL * Suboptimal weight Loss (defined as %EWL < 50% at year 1), *RWG *Recurrent Weight gain (defined as ≥ 20% gain from nadir at year 2)

At 2 years, RASG patients had a significantly lower mean weight (68.7 ± 0.8 kg vs. 79.2 ± 0.3 kg; MD = − 10.48 kg, 95% CI: −12.10 to − 8.86, *p* < 0.001) and BMI (24.7 ± 0.2 vs. 28.7 ± 0.1 kg/m²; MD = − 4.04, 95% CI: −4.45 to − 3.64, *p* < 0.001) than NRASG.

Similarly, excess weight loss (%EWL) and total weight loss (%TWL) were significantly greater in the RASG group at 2 years, with between-group mean differences of 20.81% (95% CI: 18.64–22.98, *p* < 0.001) and 8.84% (95% CI: 7.37–10.32, *p* < 0.001), respectively.

Notably, no patients in the RASG group experienced SWL at 1 year or RWG at 2 years, while these occurred in 3.3% and 5.6%, respectively, in the NRASG group (*p* < 0.001 for both comparisons).

### Comorbidity resolution and improvement

**Fig. 2:**
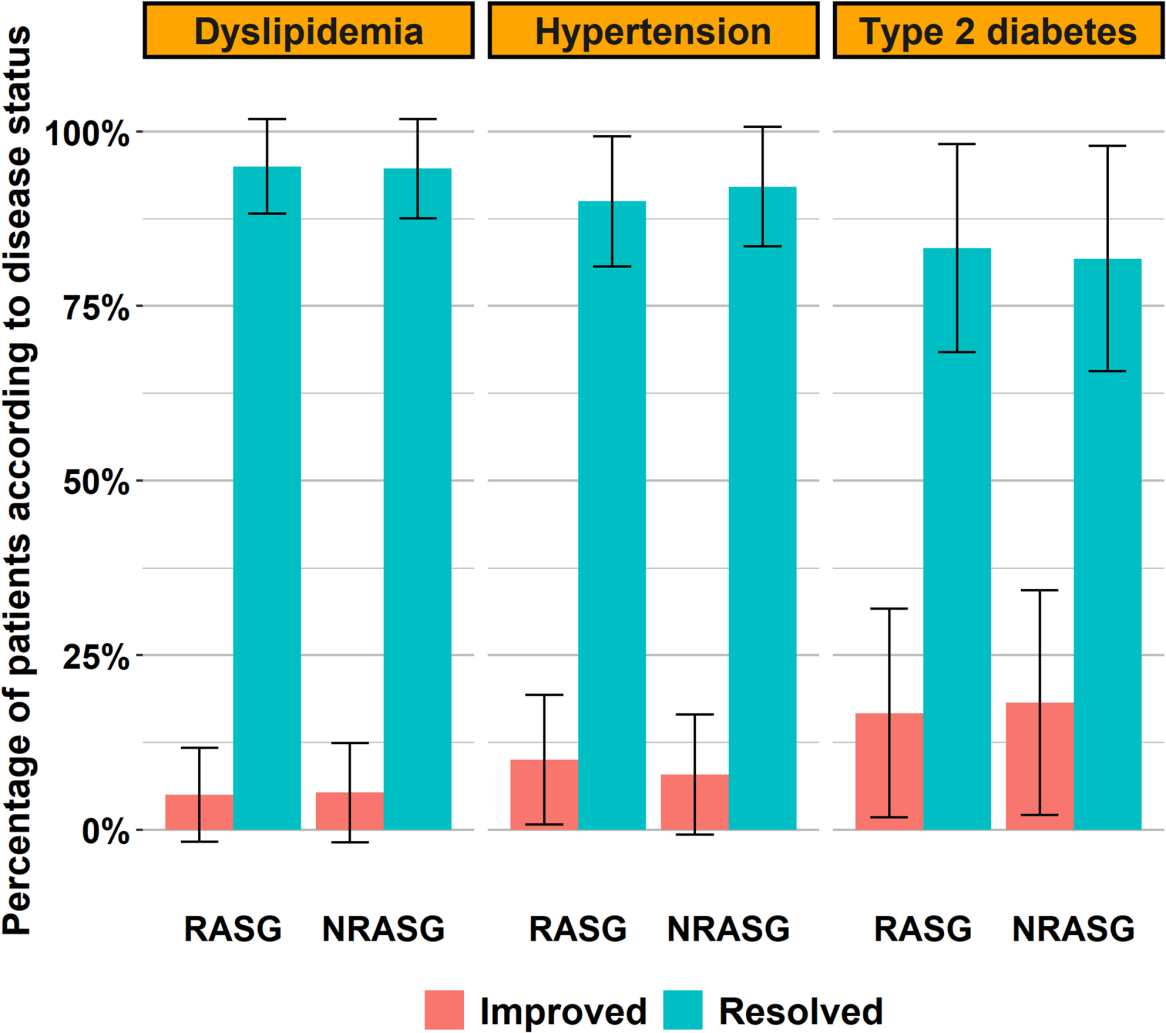
Comparison of Improvement and Resolution Rates of Major Obesity-Related Comorbidities in RASG and NRASG Groups. Bar chart showing the percentage of patients with hypertension, dyslipidemia, and type 2 diabetes who experienced either improvement or complete resolution following RASG and NRASG. Outcomes were weighted using inverse probability score weighting (IPSW). Error bars represent 95% confidence intervals. Resolution rates exceeded 90% for all three conditions in both RASG and NRASG groups. No statistically significant differences were observed between groups for any of the three conditions

**Fig. 3:**
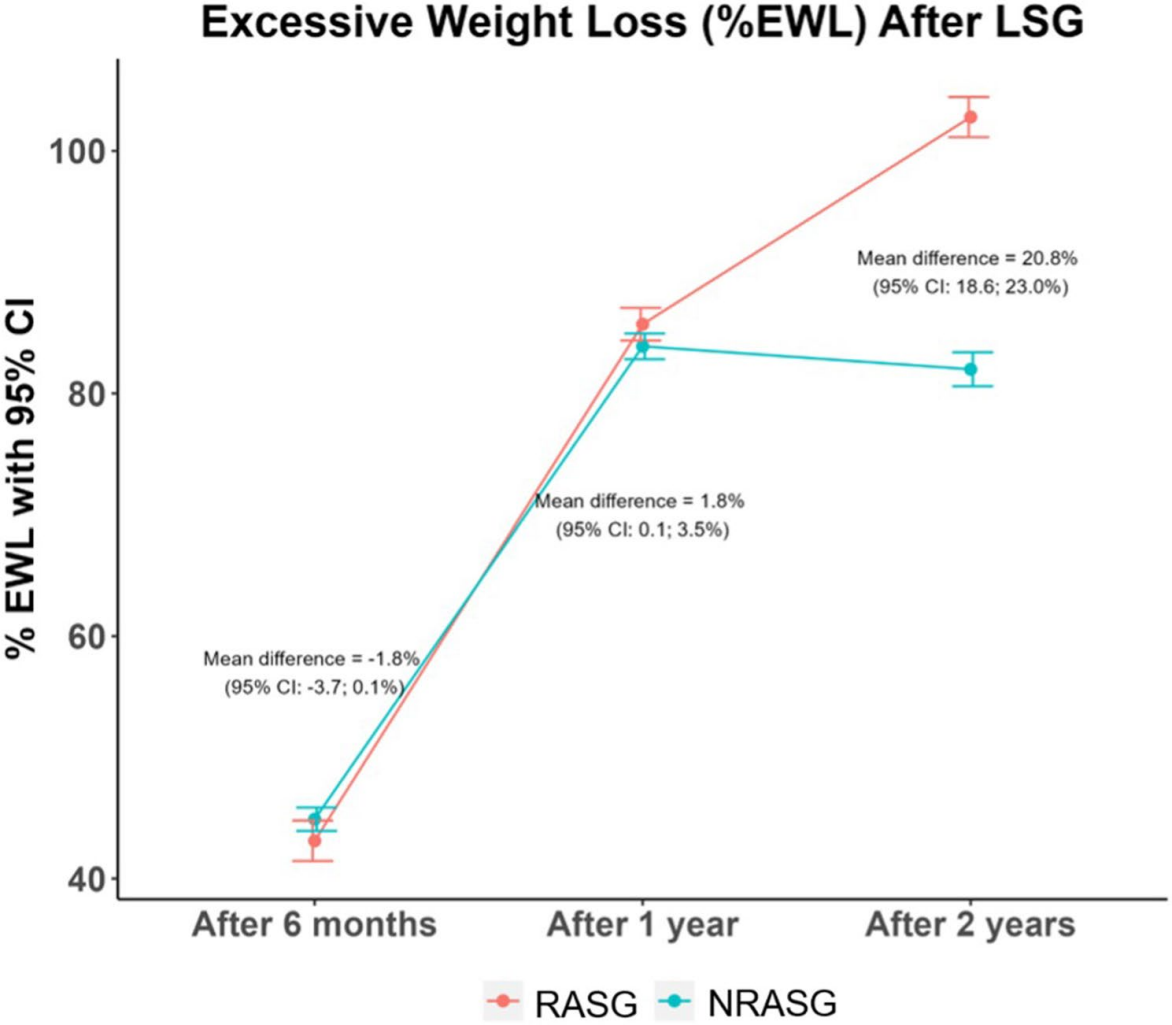
Mean %TWL over 6, 12, and 24 months postoperatively in RASG vs. NRASG

**Fig. 4:**
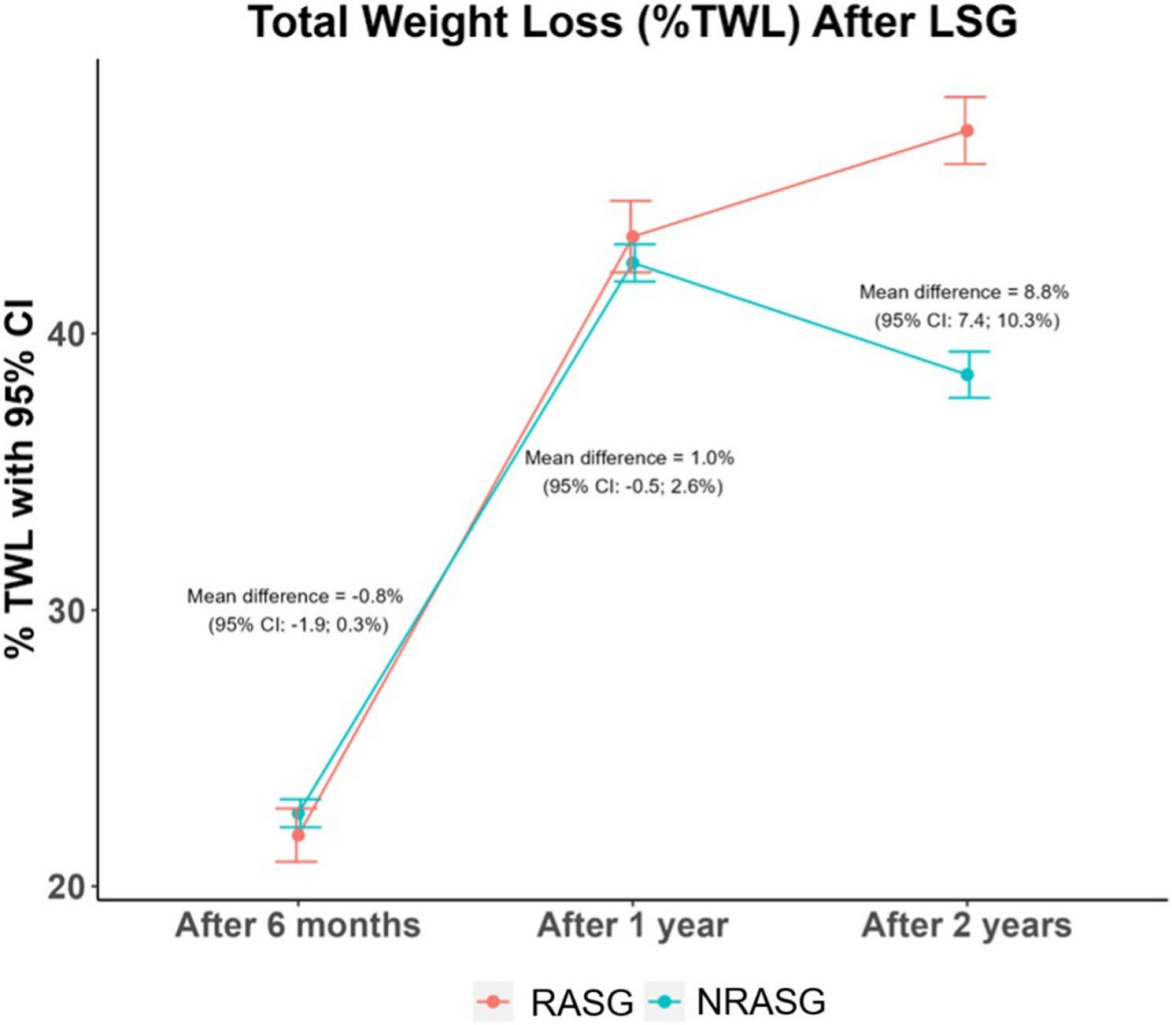
Mean %EWL over 6, 12, and 24 months postoperatively in RASG vs. NRASG

**Fig. 5:**
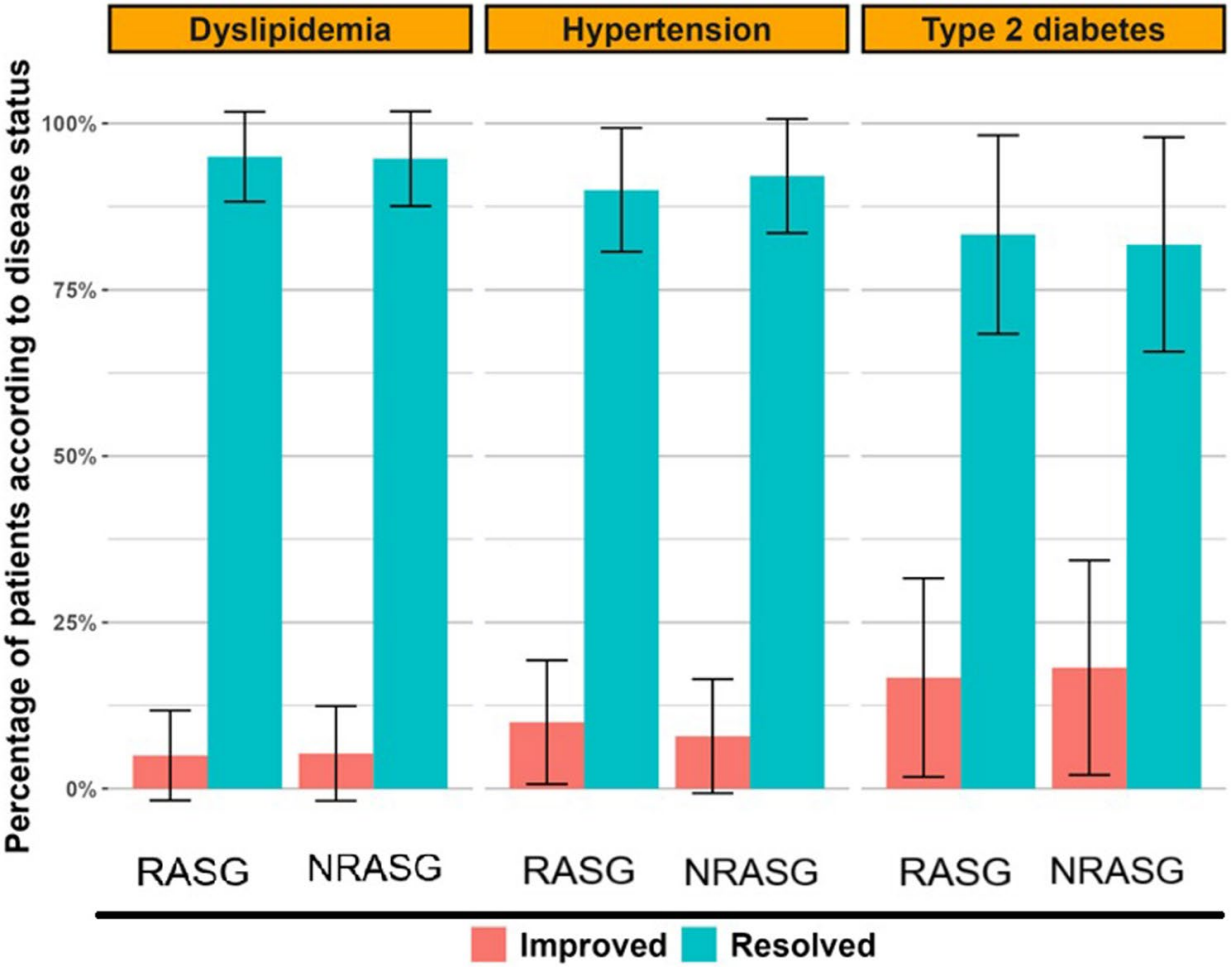
Comorbidity resolution rates by type at 24 months postoperatively

**Fig. 6:**
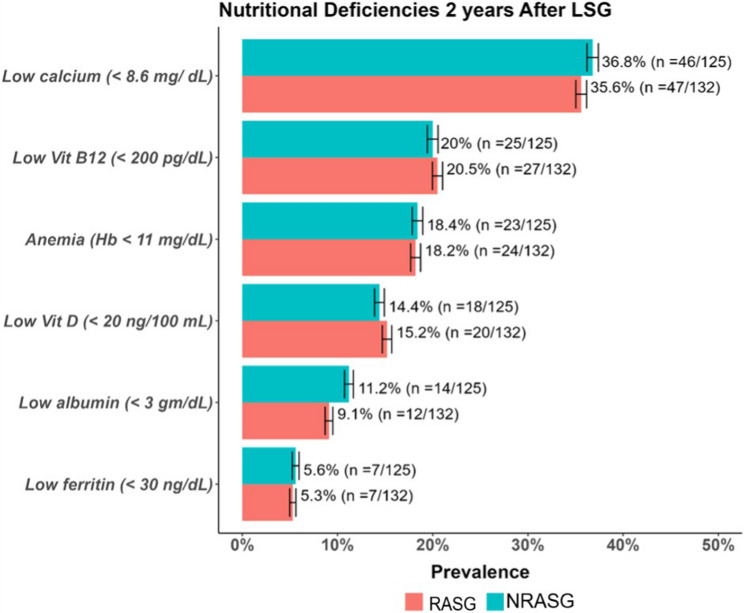
Prevalence of nutritional deficiencies at 24 months following RASG and NRASG. Common deficiencies included hypocalcemia, vitamin B12, anemia, vitamin D, and hypoalbuminemia. No statistically significant differences were observed between groups for any parameter (all *p* > 0.05)

Both groups exhibited high rates of metabolic improvement and disease resolution at 2 years, with no significant differences between RASG and NRASG (Table 4) (Figs. 2, 3, 4, 5 and 6). Hypertension was resolved in 90.0% of RASG and 92.1% of NRASG patients. Type 2 diabetes showed remission in 83.3% of RASG and 81.8% of NRASG, while dyslipidemia resolved in 95.0% and 94.7%, respectively. The remaining cases showed partial improvement.

**Table 4:** Comparison of operative characteristics, postoperative complications, and comorbidity outcomes between rasg and nrasg groups using Inverse Probability Score Weighting (IPSW)

Variable	RASG (*n*= 132)	NRASG (*n*= 125)	Test statistic	*p*
**Operative data**				
Operative time	41.6 ± 0.6	41.7 ± 0.2	t = -0.08	0.934
Added Cholecystectomy	14 (10.6)	11 (8.8)	χ² = 0.47	0.493
Endoscopy findings post-operatively at year 1				
Free	103 (78.0%)	96 (76.8%)		
GERD B	19 (14.4%)	19 (15.2%)	Fisher	0.985
Hiatal hernia	7 (5.3%)	7 (5.6%)		
Constriction at the ring	3 (2.3%)	N/A		
**Nutritional deficiencies post-operatively at year 2**				
Anemia (Hb < 11 mg/dL)	24 (18.2)	23 (18.1)	χ² = 0.00	0.990
Low ferritin (< 30 ng/dL)	7 (5.3)	7 (5.6)	χ² = 0.02	0.890
Low Vit B12 (< 200 pg/dL)	27 (20.5)	25 (19.9)	χ² = 0.02	0.885
Low albumin (< 3 gm/dL)	12 (9.1)	14 (11.1)	χ² = 0.49	0.484
Low calcium (< 8.6 mg/ dL)	47 (35.6)	46 (37.2)	χ² = 0.13	0.721
Low Vit D (< 20 ng/100 mL)	20 (15.2)	18 (14.3)	χ² = 0.06	0.800
**Comorbidity fates**				
Hypertension	*n*= 40	*n*= 38		0.574
Resolved	36 (90.0)	35 (92.1)	Fisher	
Improved	4 (10.0)	3 (7.9)		
Diabetes	*n*= 24	*n*= 22		0.926
Resolved	20 (83.3)	18 (81.8)	Fisher	
Improved	4 (16.7)	4 (18.2)		
Dyslipidemia	*n*= 40	*n*= 38		0.975
Resolved	38 (95.0)	36 (94.7)	Fisher	
Improved	2 (5.0)	2 (5.3)		

Continuous variables are shown as Mean ± Standard Error (SE); categorical variables are shown as number (%). Statistical tests include independent-samples t-test for continuous data, and chi-square or Fisher’s exact test for categorical data. Test statistics are reported where applicable. Inverse Probability Score Weighting (IPSW) was used to adjust for baseline differences

*Abbreviations*: *RASG *Ring-augmented Laparoscopic Sleeve Gastrectomy, *NRASG *Laparoscopic Sleeve Gastrectomy, *GERD-B *Gastroesophageal Reflux Disease Grade B, *N/A *Not Applicable

These findings demonstrate that while weight loss outcomes were more favorable in the RASG group, both techniques achieved similarly high effectiveness in resolving obesity-associated metabolic conditions.

### Operative data and postoperative safety

There were no significant differences between RASG and NRASG groups regarding operative time (41.6 ± 0.6 vs. 41.7 ± 0.2 min, *p* = 0.934) or rates of concurrent cholecystectomy (10.6% vs. 8.8%, *p* = 0.493), indicating that ring placement did not increase surgical complexity (Table 4).

Endoscopic assessments at 1 year revealed comparable results. The majority of patients had normal findings (78.0% in RASG vs. 76.8% in NRASG). Mild GERD (Grade B) was observed in 14.4% of RASG and 15.2% of NRASG (*p* = 0.985). Hiatal hernia and ring-site constriction were rare and not statistically different between groups (Table 4).

No cases of ring slippage, erosion, or ring removal were observed during the two-year follow-up period. Constriction at the ring was detected in 2.3% of patients (Table 4), but these were managed conservatively without surgical intervention.

These findings affirm that RASG can be performed safely with no additional operative risk compared to standard SG, when executed by experienced teams.

### Nutritional deficiency outcomes

Nutritional deficiency outcomes at two years revealed no statistically significant differences between the RASG and NRASG groups (Table 4). Hypocalcemia was the most prevalent deficiency, observed in 35.6% of RASG patients compared to 37.2% in the NRASG group (*p* = 0.721). Vitamin B12 deficiency occurred in 20.5% versus 19.9% (*p* = 0.885), while anemia—defined as hemoglobin levels below 11 g/dL—was noted in 18.2% and 18.1%, respectively (*p* = 0.990). Low ferritin levels were reported in 5.3% of RASG and 5.6% of NRASG patients (*p* = 0.890), hypoalbuminemia in 9.1% versus 11.1% (*p* = 0.484), and vitamin D deficiency in 15.2% versus 14.3% (*p* = 0.800). These findings indicate that ring augmentation does not adversely impact nutritional status when evidence-based supplementation protocols and regular postoperative monitoring are maintained.

## Discussion

Numerous studies consistently demonstrate that RASG results in significantly higher percentages of EWL% and TWL% during long-term follow-ups when compared to NRASG [18, 24, 25]. Our data reinforces these findings, showing that RASG patients not only achieve greater initial weight loss but also maintain a more favorable trajectory of sustained weight loss over time. Notably, at two years post-surgery, the RASG cohort exhibited no RWG, unlike their NRASG counterparts, who showed a discernible recurrence of weight gain. This pattern remains evident in external studies with follow-up periods extending to five years or more, indicating that RASG may offer a more enduring metabolic advantage and could mitigate the risk of RWG in selected patients [24].

The inclusion of the MiniMizer Gastric Ring^®^ in RASG fulfills a crucial biomechanical role by preventing proximal gastric pouch dilation. Over time, gastric sleeves can become distended due to factors such as dietary patterns, increased intragastric pressure, or intrinsic tissue remodeling, particularly at the gastroesophageal junction [26]. This dilation undermines the sleeve’s restrictive capacity, leading to diminished satiety signals and increased caloric intake, ultimately resulting in RWG [26].

Conversely, the ring in RASG serves as a restrictive buffer that reinforces the anatomical integrity of the gastric sleeve, effectively minimizing its distensibility and ensuring the preservation of the postoperative restriction intended by the surgical intervention. The fixed diameter of the ring also moderates the passage of food boluses, prolonging the sensation of satiety and curtailing episodes of hyperphagia [27]. Our findings, which align with other longitudinal studies, suggest that this anatomical stability may contribute to improved long-term weight maintenance in certain patients undergoing RASG, though the benefits should be weighed against the potential for ring-related complications [7, 10, 19, 22, 24, 25, 27].

MBS exerts its therapeutic impact not only through mechanical restriction but also via substantial metabolic improvements [28]. Both RASG and NRASG contribute to the resolution of multiple obesity-related diseases, including type 2 diabetes mellitus (T2DM), systemic arterial hypertension, obstructive sleep apnea, and dyslipidemia. However, the magnitude and sustainability of these improvements appear to be more pronounced with RASG [7, 25].

Specifically, the higher and more sustained weight loss achieved through RASG leads to improved glycemic control and prolonged remission of T2DM [28]. Weight loss enhances insulin sensitivity and reduces peripheral insulin resistance, mechanisms essential for reversing T2DM pathophysiology. Patients in our study who underwent RASG achieved longer-lasting reductions in HbA1c levels and had lower rates of diabetes recurrence than those in the NRASG group [7, 17].

The superior metabolic improvements observed following RASG, in our cohort, may in part reflect a more sustained reduction in visceral adiposity, which is associated with favorable changes in lipid profiles, systemic blood pressure, and inflammatory markers, as reported in prior observational analyses [16, 17]. These findings affirm that RASG not only serves a mechanical function but also acts as a potent metabolic intervention with durable systemic benefits.

The relationship between sleeve gastrectomy and gastroesophageal reflux is complex and multifactorial. NRASG has been associated with a higher incidence of de novo GERD and worsening of pre-existing reflux due to increased intragastric pressure, disruption of the angle of His, and reduced compliance of the gastric sleeve [18, 29].

Although RASG is proposed to mitigate these contributors by preserving sleeve geometry and preventing proximal dilation, our findings did not demonstrate a significant difference in GERD rates between groups at the 1-year EGD evaluation.

Mild GERD (Grade B) occurred at comparable rates in both cohorts, and no physiologic assessments (e.g., pH monitoring, manometry) were performed. Nonetheless, prior studies have reported potential reflux benefits with RASG, particularly beyond the first postoperative year [18, 29]. Future studies should not only extend follow-up beyond the early postoperative period but also incorporate objective diagnostic tools such as 24-hour pH monitoring, high-resolution manometry, and validated symptom scores to more comprehensively evaluate the impact of ring augmentation on reflux physiology.

The overall complication profiles of RASG and NRASG remain comparable, with most postoperative events being minor and manageable. Early complications such as nausea, vomiting, dehydration, or transient dysphagia are observed in both groups and typically resolve with conservative management.

However, RASG is uniquely associated with ring-related complications, including slippage, erosion, or migration. Though rare (≤ 2–4%), these events can necessitate surgical revision [20]. In the present cohort, no cases of ring slippage, erosion, intolerance, or need for ring removal were observed during the 24-month follow-up, reinforcing the short-term safety profile of the device. Despite this, no statistically significant increase in major complications or hospital readmissions was observed in our cohort or those reported in the literature [17, 30].

Due to the restrictive nature of both RASG and NRASG, patients are at risk of nutritional deficiencies, particularly in vitamin B12, folate, iron, calcium, and vitamin D. These deficiencies arise primarily from reduced intake rather than malabsorption [31]. Although there is concern that the additional restriction imposed by the ring in RASG may exacerbate these deficiencies, our data and prior studies show non-significant, comparable deficiency rates between both techniques [18, 32]. In our cohort, deficiencies such as hypocalcemia were managed with standardized supplementation, and adherence was reinforced through regular follow-up counseling and laboratory monitoring, which likely minimized intergroup differences. This highlights the importance of standardized nutritional supplementation and regular laboratory monitoring for all MBS patients, regardless of the surgical technique used.

Both RASG and NRASG are indicated for individuals with a BMI ≥ 35 kg/m² or a BMI ≥ 30 kg/m² with associated diseases [18, 30]. However, RASG may offer benefits for carefully selected subpopulations, such as patients with BMI >50 kg/m², those with prior RWG, or those at high risk of gastric dilation due to behavioral factors [14, 18, 30].

However, given the known though infrequent risk of ring-related complications requiring reoperation, its use should not be generalized to all patients. Instead, appropriate preoperative counseling and individualized decision-making are essential to determine when the potential long-term benefits outweigh the risks [7].

The adoption of RASG is well-established in high-income countries where MBS infrastructure, trained surgical teams, and postoperative follow-up programs are readily available. However, in low- and middle-income countries (LMICs), the financial cost of the MiniMizer ring and limited surgical expertise present significant barriers to implementation [18, 32].

For RASG to be adopted in LMICs, policymakers must consider value-based healthcare frameworks, including long-term cost savings and reductions in national healthcare expenditures. Investment in surgeon training and ring subsidies may yield substantial population-level benefits. Cost-effective modeling tailored to national health priorities will be essential in guiding the expansion of RASG within diverse healthcare systems.

This study’s strengths include a large sample size, uniform operative techniques by an experienced surgical team, and thorough adjustments for baseline confounders through propensity score weighting. The diverse outcome measures, including anthropometric, metabolic, nutritional, and procedural safety data, over a two-year follow-up period, add to the findings’ robustness. The integration of recent literature enhances both external validity and translational applicability.

However, the study has several limitations. Its retrospective design may introduce selection and reporting biases, affecting causal inference. Being single-centered limits generalizability due to differing institutional protocols and patient demographics. While the same surgical team performed all procedures, this may introduce surgeon-specific bias. The two-year follow-up might not reflect long-term outcomes, and there was insufficient assessment of patient adherence to lifestyle changes critical for the success of MBS. Additionally, missing patient-reported outcomes like quality of life may limit interpretation.

Notably, only patients with complete 24-month follow-up were included in the analysis. This reduces the risk of attrition bias but may limit the generalizability of the results to real-world practice, where follow-up is often incomplete. Subgroup and exploratory analyses, such as stratification by baseline BMI or time-to-event assessment of weight regain, could offer additional insights but were not feasible in our study. RWG was assessed at fixed intervals, procedures were conducted by a single experienced team using standardized protocols, and the MiniMizer^®^ ring was uniformly sized and placed. Subgrouping post-propensity score weighting could also disrupt covariate balance. Therefore, these questions should be explored in future prospective studies designed for this purpose.

This study, while conducted by the same surgical team and using similar protocols as a prior cohort [17], involves a distinct patient population operated on during 2021–2022, utilizing an updated database. This distinction is crucial for interpreting the results independently and avoiding assumptions of data duplication.

Future research should focus on prospective, randomized controlled trials comparing RASG and NRASG with long-term follow-up beyond five years. Studies should collect comprehensive data on behavioral, psychological, and lifestyle factors affecting surgical outcomes, and include stratified analyses based on baseline BMI, gender, metabolic status, and comorbidities for personalized recommendations.

Future studies should integrate economic evaluations, including real-world cost data and healthcare utilization metrics, to assess the cost-effectiveness of RASG versus standard SG, particularly in low- and middle-income countries (LMICs) with limited resources. Previous analyses indicate that lower reoperation rates and better metabolic control may economically favor RASG [29], but robust regional modeling is essential for value-based decision-making. In this regard, the upfront cost of the ring may be outweighed by potential long-term savings through fewer revisional procedures and more durable weight stability, supporting its role in value-based healthcare frameworks.

Innovation in ring design and placement should aim to reduce complications like slippage and erosion while ensuring efficacy [21, 32]. Long-term studies on patient quality of life, nutritional outcomes, and telemedicine’s role in postoperative care may provide insights for better patient support and resource efficiency.

## Conclusion

RASG provides more durable weight loss and significantly reduces the risk of recurrent weight gain compared with NRASG. Both procedures demonstrated comparable safety profiles and similar effectiveness in resolving obesity-related comorbidities. These findings suggest that adding a gastric ring reinforces sleeve integrity without increasing morbidity, supporting its role as a safe modification of standard SG. However, given the retrospective design and limited follow-up, prospective long-term studies are required to validate these outcomes and better define the role of RASG in clinical practice.

## Supplementary Information

Supplementary Material 1.

## Data Availability

The datasets generated and analyzed during the current study are available from the corresponding author on reasonable request. The cohort used in this study is entirely distinct from that of our previous publication and was derived from a separate, prospectively maintained database covering a different study period (2021–2022). No data overlap exists.

## Abbreviations

RASG: Ring-Augmented Sleeve Gastrectomy
NRASG: Non-Ring-Augmented Sleeve Gastrectomy
TWL%: Total Weight Loss Percentage
EWL%: Excess Weight Loss Percentage
BMI: Body Mass Index
RWG: Recurrent Weight Gain
SWL: Suboptimal Weight Loss
GERD: Gastroesophageal Reflux Disease
