# Supplementary material for: Evaluating The Impact of Ring Augmentation In Sleeve Gastrectomy: A Retrospective Propensity-weighted Cohort Study
Source: BMC Gastroenterol. 2025 Dec 2;25:857. doi: 10.1186/s12876-025-04476-9 (PMC12690876; doi:10.1186/s12876-025-04476-9)
Supplement: Supplementary file 1 — Supplementary Material 1. [file 12876_2025_4476_MOESM1_ESM.docx]

**Supplementary Material**


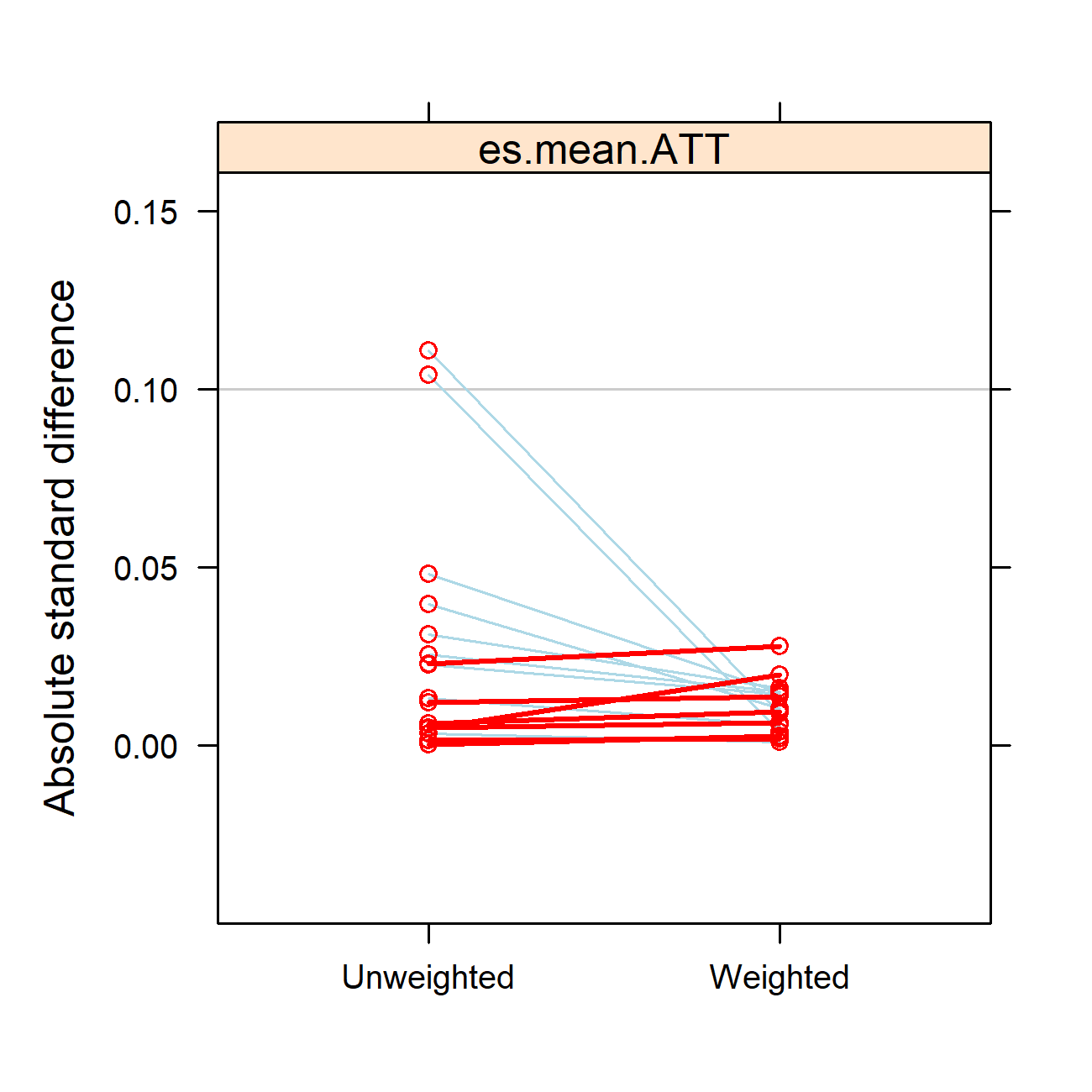


**Figure S1: Balance of Baseline Covariates Before and After Propensity Score Weighting.** Absolute standardized mean differences (SMDs) for all baseline covariates between the RASG and NRASG groups before (unweighted) and after (weighted) application of propensity score weighting using the average treatment effect on the treated (ATT) approach. Each line connects the same variable's SMD before and after weighting. All post-weighting SMDs were reduced below the conventional threshold of 0.10, indicating successful covariate balance.


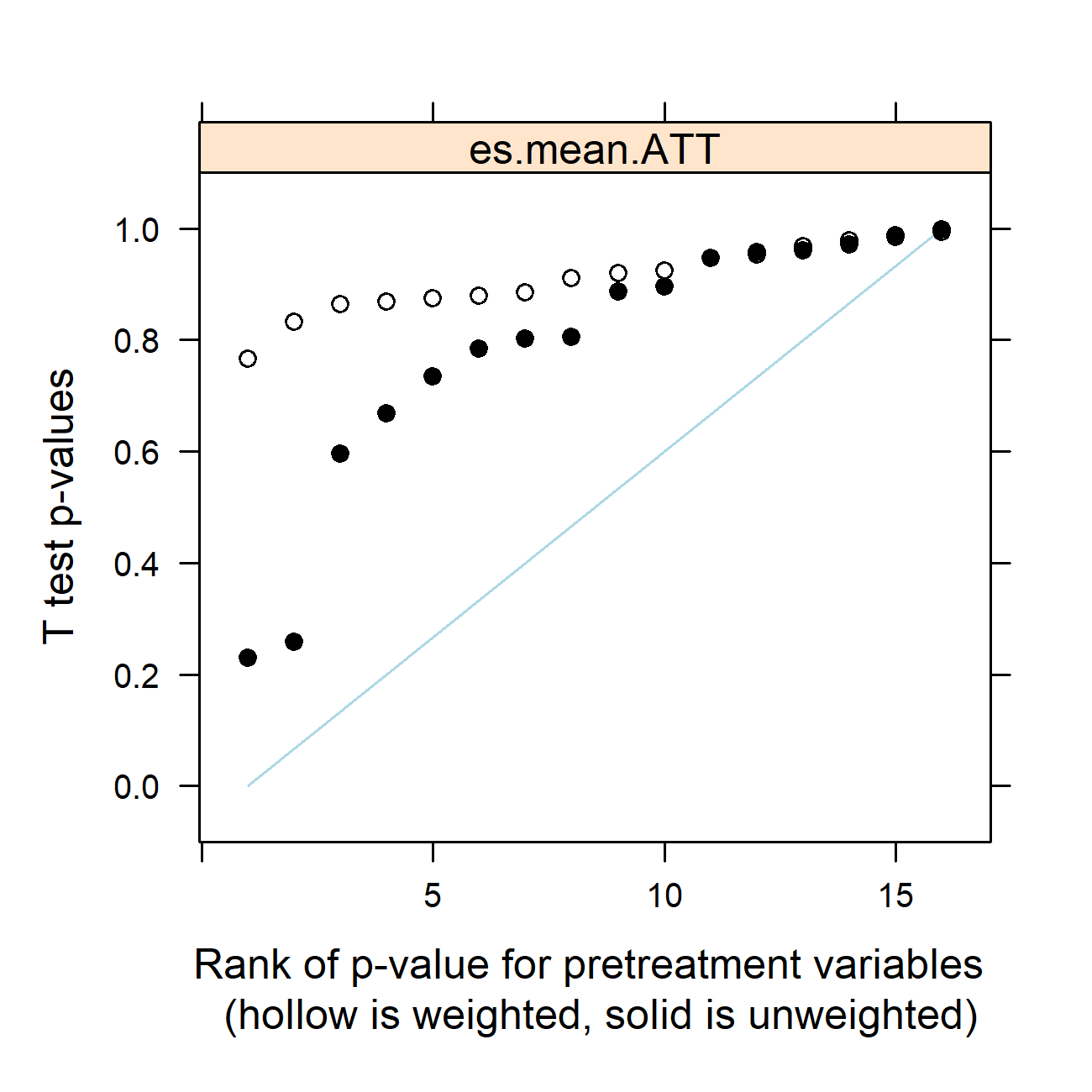


**Figure S2: Improvement in Covariate Balance Based on Ranked p-values Before and After Weighting.** Ranked p-values from t-tests comparing baseline covariates between RASG and NRASG groups before (solid black circles) and after (hollow circles) applying propensity score weighting using the average treatment effect on the treated (ATT) framework. Post-weighting p-values shifted upward, reflecting improved covariate balance and reduction of baseline differences.
